# Neuroplasticity as a Foundation for Decision-Making in Space

**DOI:** 10.3390/neurosci3030033

**Published:** 2022-08-09

**Authors:** Margaret Boone Rappaport, Christopher J. Corbally

**Affiliations:** 1 The Human Sentience Project, LLC, Tucson, AZ 85704, USA; 2 Vatican Observatory, Department of Astronomy, University of Arizona, Tucson, AZ 85721, USA

**Keywords:** neuroplasticity, plasticity, decision-making, deconditioning, microgravity, human neurology, space exploration, space medicine, mission success

## Abstract

This is an exploratory review of two very recent, intersecting segments of space science: neuroplasticity in space, and decision-making in space. The high level of neuroplasticity in humans leads to unfortunate neurological and physical deconditioning while the body adjusts to the new space environment. However, neuroplasticity may also allow recovery and continued functioning of decision-making at a level necessary for mission completion. Cosmic radiation, microgravity, heightened levels of carbon dioxide in spacecraft, and other factors are being explored as root causes of neurological and physical deconditioning in space. The goal of this paper is to explore some of the lines of causation that show how these factors affect the capacity of humans to make decisions in space. Either alone or in groups, it remains essential that humans retain an ability to make decisions that will save lives, protect equipment, complete missions, and return safely to Earth. A final section addresses healthcare, medical intervention, and remediation that could help to “harness” neuroplasticity before, during, and after spaceflight. The dual nature of human neuroplasticity renders it both a cause of problems and also potentially the foundation of remediation. The future of research on both neuroplasticity and human decision-making promises to be full of surprises, both welcome and otherwise. It is an exciting time in research on space medicine.

## 1. Introduction

### 1.1. Will Neuroplasticity Help or Hinder Decision-Making, or Both, and How?

This is an exploratory review of two very recent, intersecting segments of space science: neuroplasticity in space, and decision-making in space. The high level of neuroplasticity in humans leads to unfortunate neurological and physical deconditioning while the body adjusts to the new space environment. However, neuroplasticity may also allow recovery and continued functioning of decision-making at a level necessary for mission completion. Cosmic radiation, microgravity, heightened levels of carbon dioxide in spacecraft, and other factors are being explored as root causes of neurological and physical deconditioning in space. The goal of this paper is to explore some of the lines of causation that show how these factors affect the capacity of humans to make decisions in space. 

An analysis of research on a topic as complex as human decision-making in space requires limitations to be set, and we have confined our review to articles in the past several years or references that illuminate broad issues. Sasmita et al. [1] wrote of clinically “harnessing neuroplasticity”. This type of health management—making the best of the high level of human neuroplasticity, treating unfortunate sequelae, anticipating the need for health management *en route* to distant locations, and preparing for neurological changes before spaceflight—may well be the key to survival on long space voyages, and essential to human exploration and population of the solar system. It is possible to envision a time when human neurological changes in spaceflight will be routinely anticipated and managed with appropriate care. Section 5 of this article describes types of remedial programs, both existing and proposed, but it is not an exhaustive list because the nature of human neuroplasticity is still being discovered.

Readers are cautioned that research studies on both neuroplasticity in space and decision-making in space are still in an early phase, and results are often best stated generally in terms of identifying which neurological tissues are affected and which are not. Important studies are described here concerning the effects of space, which could theoretically have equally important effects on decision-making. However, answers as to “How much?” “When?” “Why?” and “Is the change reversible?” are, in most cases, questions yet to be answered by future research. Many questions are posed over the following pages that do not yet have firm answers; this is done to (1) suggest to readers certain avenues for future inquiry, help them frame research questions, and help them decide which aspects of deconditioning require medical intervention the most urgently, (2) give a sense of how fast-moving research in space neuroscience is proceeding, and (3) caution readers that final answers are not always available. We undertook this research aiming to determine how dangerous and difficult human neurological change will be for longer spaceflights to Mars and asteroids. There is some concern about whether humans will be able to safely make such journeys, and there are rightful reasons for that concern, as will become apparent.

### 1.2. Decision-Making in Space: The Issues

Shelhamer and Scott [2] provided an inclusive overview of the biomedical issues awaiting humans in space as we venture beyond the Moon to Mars, asteroids, and farther:

“The major biomedical issues common to all destinations and mission types considered here include space radiation exposure, life support including breathing air contamination, pressure-suit performance and decompression sickness, physiological and cognitive effects and their monitoring, crew autonomy, and medical concerns. This is a subset of the larger set of issues that NASA has currently identified as the primary risks to humans during long-duration spaceflights…” (p. 800).

Their summary mentioned a variety of biomedical issues, some of which are addressed below, such as nutrition and immune system changes in reaction to spaceflight. Decision-making was not listed specifically, perhaps because little is known about decision-making in space. In the literature on neuroplasticity and decision-making, these two omnipresent human traits were linked only very recently at the cellular level, e.g., as in Popova et al. [3], and behaviorally [4,5].

Among the best available reviews of cognitive performance in spaceflight (a category that must eventually include decision-making) are those by Strangman and colleagues [6,7]. Their summaries mention factors that could impact human decision-making in space, such as radiation, varying levels of CO_2_ (explored in Section 2), dehydration, chronic stress, isolation, and others. The reviews included the outcomes of tests on a variety of cognitive capacities and maneuvers, which could affect specific decision-making processes. Strangman and colleagues wrote that the effects during the early period of spaceflight involve “…reductions in motor speed and accuracy, some perceptual deficits, impairments in attention switching, and emotional interference in cognitive decision-making [6,8]” [7] (pp. 402–403).

A more inclusive view of the complex process of human decision-making is still difficult to describe holistically, sequentially, and precisely, and it is equally difficult to operationalize features in space or analog research and gather meaningful results for an impression of which factors most need medical attention and, therefore, threaten mission success. The nature of neuroplasticity and the human high level of that trait confound the picture of decision-making because there is evidence that some neurological changes begin to stabilize after weeks of spaceflight. This hopefully suggests changes might eventually be “overcome” to an extent. In the future, when decision-making in space is more thoroughly studied, results could show that it is not irreparably affected in spaceflights lasting years. That does not mean the human body is not stressed or altered, but that crew might press through in difficult circumstances and continue to make good judgments. The question then becomes: for how long?

The perspective taken here is that human decision-making is a process (fast or slow) that occurs at the genetic, synaptic, hormonal, organ, network, behavioral, and group (i.e., crew plus mission control staff) levels. It includes all the components of Strangman and colleagues’ scheme: perception, motor, memory, attention, spatial transformation, “complex”/operational, executive function, emotional processing, and social processing [7], plus non-cognitive variables.

Social stimulation is important for good decision-making of many types. ICE studies in “Isolated, Confined, and Extreme” environments (such as those on a spacecraft) show that social isolation affects decision-making and the functioning of the entire “crew”. There is much to suggest that human group problem-solving is unique in the animal world, in part due to its inclusion of symbolic systems. The process becomes apparent at around 3 or 4 years old, when youngsters begin to solve problems in groups [9]. It is not a smooth and uncontentious process as they try one thing, then another. Children disagree; adults disagree. In comparison, this process is not evidenced by other primates. Group problem-solving is one of the best traits humans have at their disposal now to survive spaceflight. In the future, with the introduction of artificial intelligence, better results may be achieved with a combination of human and AI dual-group decision-making.

### 1.3. Neuroplasticity in Space: The Issues

There is an emerging, but incomplete, understanding of both plasticity and neuroplasticity as biological traits, which occur at a relatively high level in humans compared to other species, even extinct species in the genus Homo [10]. In a review of gene function in the unique NASA [the U.S. National Aeronautics and Space Administration] Twin Study, Garrett-Bakelman et al. gave results on the broad nature of human biological plasticity. They wrote that, “primary immune functions, including chemotaxis, antigen distribution and trafficking, and presentation through the lymphatic system were maintained. Overall, their data show plasticity and resilience for many core genetic, epigenetic, transcriptional, cellular, and biological functions” [11] (p. 15). The key is *resilience*. Neuroplasticity causes problems, but it also enables resilience.

Neuroplasticity underlies a range of neurological changes in human spaceflight, from the synaptic level to the main regulators of brain neuroplasticity [3], to reorganization of the adult brain [12,13], to changes in broad systems such as the immune system, as studied in mice [14], and especially, as one might expect in the absence of gravity, changes in the human vestibular system—from the sensory organs to the Purkinje cells of the vestibular cerebellum [15]. The vestibular system and its resulting “orthostatic intolerance” was one of the first neurological changes to be linked to “microgravity-induced plasticity” [16].

Correia [17] reviewed “neuronal plasticity” of the vestibular system, and the words he chose—adaptation and re-adaptation—signaled an emerging understanding of the *dual nature of neuroplasticity*. It is “adaptive” when changes occur in response to space. More specifically, it is both “adaptive” and troublesome—which appears odd. However, if “adaptations” remain when crew return to Earth, they can be “maladaptive”. Gradually, a more general concept of dual-valence “neuroplasticity” has appeared. It is difficult to use the word “adaptive” because what is adaptive in one gravity is not so in another gravity. Newberg [18] gave a good summary of vestibular changes both in space and those anticipated in partial gravity on the Moon. They are potentially quite troublesome, both in space and on Earth, and potentially on the Moon, Mars, and farther out in the solar system.

The theory of the nature and evolution of human neuroplasticity has been accelerating in the past ten years. Hrvoj-Mihi et al. [19] emphasized that plasticity refers to both functional and anatomical changes—“from molecules to bones”—and that much of human neuroplasticity emerges because of heterochrony, i.e., change in the timing or rate of development relative to an ancestor. In the same year, 2013, Gómez-Robles, Hopkins, and Sherwood [20] emphasized human evolutionary features related to what later became known as “exaptation” of one side of the brain tissues to accomplish evolutionarily new cognitive tasks. Without a pervasive neuroplasticity, human accommodation of new and complex cognitive tasks, which often arise as part of decision-making in spaceflight, would be more challenging. Adaptation, re-adaptation, recovery, and repeated recovery can all occur, but it is not yet known how fast. The cohesion of human neurological processes is often maintained even in environments of cosmic radiation or heightened CO_2_, and in various gravities. The overall ability of the human neurological system to restabilize and function is quite remarkable. It gives us hope that with careful medical management, the high-level decision-making that sets the human species apart could be maintained in the currently anticipated gravities.

### 1.4. The Decision Environment in Space: What It Feels Like

The human decision environment in space is complicated so let us provide a sense of how decision-making in space *feels*. The following excerpt is an historical example of decision-making in space documented by physician and astronaut David A. Wolf, M.D., who arrived at the space station Mir for a stay of 128 days on 25 September 1997. During his stay, he engaged in a spacewalk (extravehicular activity, EVA) of over 41 h, working with space veteran Commander Anatoly Solovyev. Wolf described the tense action inside Mir while docking a supply ship with the Russian space station. We hope this short excerpt from a long diary will give readers a feeling for decision-making in space.

*“It was almost eerie to see the robot ship loom in out of the darkness. The view from Anatoly’s [Solovyev] tele-operated pilot station was as seen by the cargo ship, closing in on this amazing space station. Its computer mind correcting for errors in the cross-hairs on the docking target, just as Anatoly would have done himself. It behaved almost human. Anatoly’s hands were lightly poised on the remote control sticks, ready to manually take over at the first sign of bad decision-making by the computer pilot.*

*“He and Pavel [Vinogradov] checked approach speeds and positions from the console. In their minds they had transported themselves and were sitting in the cargo ship…. As I watched their moves and words [and saw] how confidently they worked together from training and experience, my few thoughts of what happened to Mike Foale a few months ago were quenched.” Then, “Thunk,” the Progress docked. “It hit pretty firmly—which is normal. No pressure sensations in my ears. Docking mechanism properly engaged. The silence of tuned nerves was broken by laughter and handshakes. Supplies had arrived”* [21].

## 2. Methodological Creativity Is Essential for Space Neuroscience

In this early stage of research, it is important to acknowledge the creativity that has already influenced the development of research designs and methodologies to study neuroplasticity in spaceflight—especially as it is related to something as complex as decision-making. To review the panoply of methods used in just the citations in this paper would generate an entire extra paper. Several of the more creative techniques are listed here. For example, there is a centrifuge that simulates gravity for mice on the International Space Station (ISS) [14,22,23]. This helps to determine how long it takes for “deconditioning” to reverse, if at all.

New analytic tools were explored by Doroshin and colleagues [24], who followed Van Ombergen et al.’s [25] review of fMRI [functional magnetic resonance imaging] studies with a study of twelve cosmonauts on the ISS using *d*MRI—*diffusion* magnetic resonance imaging—useful in the study of the brain’s white matter. Their research sought “to use fiber tractography to investigate which specific tracts exhibit structural changes after long duration spaceflight and may direct future research to investigate brain functional and behavioral changes associated with these white matter pathways” [24] (p. 1). They found “significant microstructural changes in several large white matter tracts, such as the corpus callosum, arcuate fasciculus, corticospinal, corticostriatal, and cerebellar tracts” [24] (p. 1). This type of research points to important brain changes and lays a foundation for studying decision-making in space. Future studies from these researchers and others will likely produce results that clarify the importance of these changes in the white matter.

Methodologically, Popova and colleagues’ [3] finely detailed research design for studying the regulators of brain neuroplasticity was among the best. Their results showed which regulators are affected by spaceflight. The researchers analytically teased apart the neurotransmitters and neurotrophic factors most affected by space, and then, which brain tissues are most affected, and whether through dopamine or serotonin pathways. They concluded that long-term spaceflight affects “genetic control of both neurotransmitters and neurotrophic factors, although the sensitivity of various systems [BDNF, GDNF, and CDNF^1^] is different. The effects on serotonin and dopamine pathways are also different, which is important because dopamine is widely connected to reward, aversion, and decision-making. There is concern that dopamine deficiency in spaceflight could well affect motivation and decision-making” [3] (pp. 401–403). As such, the sensitivities of brain tissues can be understood to differ. In Section 4.2 below, Popova and colleagues’ work on “risk neurogenes” (as opposed to “spaceflight resistant genes”) is summarized.

This type of analysis delves deeply into the potential connections between neuroplasticity and decision-making. It is not a simple picture at the genetic and regulatory levels, and neither is it simple at the level of humans deciding how to maneuver the docking of a re-supply vehicle to a space station. Creative research designs will be needed to connect one level of neurological complexity to another, and then, to various stages of decision-making. Even at this very early phase of research, the risk associated with an absence of good decision-making pushes research ahead.

Our collection of data series for the study of humans at various levels of gravity and microgravity will be enhanced by Artemis, a lunar base camp at the South Pole of the Moon, and Gateway, a lunar orbiter. With human subjects in three environments—the Earth at full gravity, the Moon at 1/6th, and Artemis at virtual weightlessness—it will be possible to construct data series of many types. Measurements with three points are often more useful than just two.

Human experience in space is limited, and much research is from analog research settings that simulate the effects of weightlessness. “Long” experiences in space of a year or half-year for humans and rodents pale in comparison to the years that voyages will take in the future. Little is known about human health and survival on voyages that will take years, such as to Mars or asteroids. Some information is known about human life in gravities as low as the Moon’s (1/6th g) or Mars’ (1/3rd g) [26]; still, further work is needed to try to anticipate the neurological consequences of lengthy spaceflight and microgravity. Mhatre et al. [27] wrote in anticipation of longer voyages that “determining neurobiological and neurobehavioral responses, understanding physiological responses under Central Nervous System (CNS) control, and identifying putative mechanisms to inform countermeasure development are critically important to ensuring brain and behavioral health of crew on long duration missions” [27] (p. 908). Countermeasures are reviewed in Section 3 of this article.

It is important to acknowledge that the research results to date are not trivial. They may be overcome by remedial measures, but at this early date, the neurological changes do not appear inconsequential. Mhatre et al. [27] provided results showing that “exposure to cosmic radiation such as ^4^He, ^16^O, ^48^Ti, causes significant reduction in dendritic complexity, spine density, altered spine morphology, and synaptic integrity, along with the increased expression of postsynaptic density protein-95 (PSD-95) in the medial prefrontal cortex [28,29], *a region associated with decision-making and retrieval of long-term memory* [emphasis added]” [30] (p. 917). In other words, there is already evidence, even at this early juncture, that cosmic radiation affects brain tissues associated with decision-making.

## 3. Decision-Making in Space: Lines of Causation

In this section, we describe some known lines of causation from environmental variables to decision-making outcomes in spaceflight. Some research considers the interactive effects of two variables concurrently, as in Mahadevan et al. [31]. It should be noted that many of these studies define “human decision-making” differently from one another. The routes of causative factors from a spacefarer’s environment, experience, behavior, and biology to a decision outcome can be complex, even if an ultimate decision involves strictly technical choices without social or emotional involvement. Sequences of decisions in finely tuned mechanical tasks may also go on to affect later decisions and tasks. The decision environment in space is complex, and it is not likely to become less so until well into the future.

A fascinating body of literature is emerging that delves deeply into the interactive neurology of different brain tissues in the process of human decision-making (i.e., making a choice), for example, interaction between the prefrontal cortex and the hippocampus [32]. At the same time, there are research mushrooms on the physiological and neurological effects of spaceflight in studies on rats and mice. NASA award-winning work by Shirakawa and colleagues with a mouse habitat cage demonstrated gravity effects and the reversal of deconditioning [14,22,23]. Horie and colleagues’ work [14] using a centrifuge with mice on the ISS provided research results demonstrating the effects of microgravity on the vertebrate immune system, followed by recovery from these effects with the use of a centrifugation cage simulating gravity. Microgravity causes the thymus to atrophy, through a reduced proliferation of thymic cells. The authors suggested that “exposure to 1× *g* might alleviate the impairment of thymus homeostasis induced by spaceflight” [14] (p. 1). It is conceivable that a centrifugal apparatus could be developed for humans. It would be a challenge for design engineers, but perhaps well worth it.

Shelhamer rightly cautions that “animal experiments will be of great value in elucidating partial-g effects, but they have uncertain transfer to human responses… The reduced but non-zero gravity level on the moon may well be sufficient to halt or dramatically reduce the main aspects of physiological deconditioning seen in weightlessness” [26] (p. 117). There is also hope that artificial gravity and/or conditioning programs could help to reduce or reverse the negative effects of microgravity in spaceflight, and in low-g on the Moon and Mars, including cognitive factors that affect decision-making.

“Deconditioning” in spaceflight happens in the guise of changed brain tissues, muscles, reduced balance and motor control, and fluid shifting toward the head. It is not only a set of changes to human neurology, but neurological deconditioning concerns space program planners because of its effects on decision-making. How surely can mission success be guaranteed? How much of the inherent riskiness of space exploration can be reduced?

### 3.1. Effects of Spaceflight on Decision-Making: Example of CO_2_

#### 3.1.1. Improvement in Decision-Making in Dangerous Circumstances

This section addresses one of the clearest lines of causation emerging from research results to date on the decision environment in space. There is a well-documented build-up of CO_2_ gas in spacecraft interiors, and there are important research results showing that the CO_2_ concentration does indeed affect decision-making. To date, most experimental examples of human decision-making involve relatively simple cognitive and motor tasks, although Earth-based research can involve more complex scaled measures [33]. In the future, assessment of decision-making will grow in complexity from changes at the neuronal level to the regulatory level, to localized changes in the brain, and to behavioral outcomes of human decision-making. At every juncture, gravity, the internal environment of a spacecraft, and the social involvement of the crew could all impact critical decisions.

Research results show that ambient CO_2_ affects human performance in a dual cognitive-motor task in a spaceflight analog (head-down-tilt bed rest, HDBR). The addition of CO_2_ better simulates conditions on the ISS [31]. These findings follow research results by Satish et al. [33] on observed, Earth-based indoor performance decrements even after exposure to low-to-moderate CO_2_ concentrations. At higher concentrations, large and statistically significant reductions in performance occurred on seven of the following scales of decision-making performance: basic activity level, applied (opportunistic) activity, focused activity, task orientation, initiative (new activities), openness to information search, information usage, breadth of approach (flexibility), and basic strategy (number of strategic actions) [33] (p. 1674).

Performance on one scale—focused activity—*increased* in the presence of higher concentrations of CO_2_ [33]. “Focused activity” was described as “strategic actions in a narrow endeavor” (p. 1673), and results were in some ways similar to the results for “information search”, described as “openness to and search for information” (p. 1673). The authors noted, “most decision-making variables showed a decline with higher concentrations of CO_2_, but measures of focused activity improved. Focused activity is important for overall productivity, but high levels of focus under nonemergency conditions may indicate ‘overconcentration’” (p. 1675).

It is clear from results on different scales that decision-making performance is not a singular concept. So, it should be split into components for research on humans in spaceflight. It should also be noted that it is precisely “focused activity” that may be critically needed in emergency situations during spaceflight. To be able to continue to perform, or even improve your performance, in conditions that are not ideal is a very handy human ability when danger is present or decision-making entails life or death. This ability to perform better in crises suggests a flexibility in neurological functioning that toggles between decision-making in slow/safe circumstances and that in fast/dangerous circumstances.

#### 3.1.2. Inconsistent Neurological Changes Point to “The Right Stuff”

Neuroplasticity implies, if anything, a range of human responses to environmental change. For example, the results on the effects of ambient CO_2_ on decision-making are not consistent. Scully et al. [34] suggested that the environment of space may not be the same for all individuals. In their double-blind crossover study with 22 participants at the Johnson Space Center, each participant was randomly assigned to one of four groups depending on the CO_2_ concentration (600, 1200, 2500, 5000 ppm). Scully et al. reported results that, in “astronaut-like” subjects: “There were no clear dose–response patterns for performance on either SMS [Strategic Management Simulation] or Cognition. Performance on most SMS measures and aggregate speed, accuracy, and efficiency scores across Cognition tests were lower at 1200 ppm than at baseline (600 ppm); however, at higher CO_2_ concentrations performance was similar to or exceeded baseline for most measures. These outcomes, which conflict with those of other studies, likely indicate differing characteristics of the various subject populations and differences in the aggregation of unrecognized stressors, in addition to CO_2_, are responsible for disparate outcomes among studies” [34] (p. 1). They suggested “longer exposure durations to verify that cognitive impairment does not develop over time in crew-like subjects” [34] (p. 1).

It may be that self-selection and other-selection factors may have operated in this and comparable studies. Test group differences suggest that there may be something to the expression “the right stuff”, with some of that quality relating to the ability to make good decisions when impaired. This can only be determined through guesswork based on the reactions of test pilots, Special Forces trainees, and some average citizens in a time of disaster, such as a volcanic eruption. Few humans have been challenged quite so much as in a space environment. Therefore, questions about the effects of CO_2_ remain. Why did the performance of “astronaut-like” subjects not deteriorate in the research by Scully et al. [34]? Did subjects make an effort so that their performance did *not* deteriorate? Are individuals in space programs or similar particularly able, like test pilots, to experience environmental effects and circumvent them when they need to? Such questions need full exploration. However, it will come as no surprise to anyone that some humans can tolerate space better than others.

#### 3.1.3. Neuroplasticity-Based Adaptation and Recovery: What Is Their Duration?

Let us trace the lines of causation between ambient CO_2_ and decision-making, and then we will discuss neurological recovery in light of the high human level of neuroplasticity.

Salazar and colleagues rightly notes that, “astronauts on board the International Space Station (ISS) must adapt to several environmental challenges including microgravity, elevated carbon dioxide (CO_2_), and isolation while performing highly controlled movements with complex equipment” [35] (p. 1). The researchers initially demonstrated that visuomotor adaptation indeed occurs in the short-term during a spaceflight analog situation with HDBR and elevated CO_2_. They then examined concurrent brain activity using fMRI scans [35,36] and verified the effects on neural correlates of visuomotor adaptation [35]. After initially demonstrating visuomotor adaptation, they verified changes in temporal and subcortical brain regions in early adaptation, and then, in the right fusiform gyrus and right caudate nucleus. This demonstrated, as many fMRI studies in this field do, that changes and recovery occur in brain tissues; they are not superficial and sensory.

Yet, the question remains: do these changes in short-duration spaceflight or analogs suggest further adaptation and/or further recovery on very long spaceflights lasting years? Will space crew achieve a “new normal” or a functional adaptation that works well in the environment of space? Most intriguing: will the adaptation persist once spaceflight ends, and how much does that matter? Humans who go on long space journeys may come to expect a certain amount of permanent neurological change. The NASA Twin Study [11] hints that this may be true.

#### 3.1.4. A Hopeful Possibility: The Brain May Adapt and Recover over Time

In a HDBR spaceflight analog research setting, with and without CO_2_, Mahadevan et al. [31] delved into the effects of a simulated spaceflight environment on dual tasking—a major issue for space crew, especially in emergencies or difficult maneuvers. The study design included both single and dual tasking, using one cognitive and one motor task.

Mahadevan et al. [31] recalled Manzey et al. [37], Manzey and Lorenze [38], and Hupfeld et al. [39] in describing a decline in dual-task performance after astronauts begin spaceflight, but then, they begin to recover in space. They reported changes in structural brain metrics in the cerebellum, for example, which were greater after a short spaceflight than after a longer one. “This could indicate that the brain accommodates to the novel environment of spaceflight and begins to return to its normal structure and physiology during longer flights” [40] (p. 13).

That is a hopeful possibility, and if true, it is a striking example of neuroplasticity’s capacity to support humans in space. It is a type and level of adaptive ability that will serve humans well as they fan out across space on short and longer voyages. Mahadevan and colleagues’ results appear to reflect an ability of the human spacefarer to adapt and return to normal functioning, even in challenging circumstances, i.e., microgravity and elevated CO_2_. They found clear patterns of recovery back to the baseline after HDBR + CO_2_-induced change [40] (pp. 10–11). Questions remain, however, about which neural tissues recover at which rates, and what the implications are for human decision-making.

### 3.2. Role of the Hippocampus in Decision-Making: A Prime Target for Space Research

One of the most important areas for future research on decision-making in space is an examination of the interaction between the hippocampus and the prefrontal cortex in human decision-making. However, as of the time of writing, this research has not yet been reported in either a human or rodent model for the space environment. Using a rat model, Tang et al. reported experimental results on the hippocampus-prefrontal cortex cooperative interaction at different neurological timescales (slow, fast), to support decision-making [32]. Hippocampal neural activity was seen to coordinate with prefrontal activity that predicted upcoming choices. However, this research did not take place in the context of space or in a space analog situation. Results that show this occurs in space for astronauts will hopefully be forthcoming. As previously noted, exposure to high-energy space radiation in rodents resulted in memory deficits [13]. Therefore, there is good reason to pursue this research thread. The nature of the complex signaling between the hippocampus and prefrontal cortex in memory-based decision-making has only recently come to light, but again, it is not clear at this point how a space environment will affect results [32]. There is much to learn to improve the safety of a crew on a Mars mission, or a crew settling at the Artemis lunar base camp at the Moon’s South Pole, who will also be exposed to radiation from space.

## 4. Risk, Neuroplasticity, and Decision-Making

The inherent risk of human spaceflight and later settlement on the Moon, Mars, asteroids, and the moons of Jupiter and Saturn cannot be overestimated. Human species have ventured far from “home” in exploratory pursuits that began long before the line evolved into *Homo sapiens*. These tendencies to wander were clearly present in modern humans’ predecessor, *Homo erectus*, who trekked from Africa to all the way to Southeast Asia. Now, *Homo sapiens* is set to embark on its next great exploratory venture: space and settlement of the solar system, and perhaps beyond. It will be dangerous, and many people will die. In that context, it is important to understand what “risk” means—perhaps danger, perhaps adventure, perhaps escaping one’s past, and surely, by lassoing near-Earth objects (NEOs) as sources of water and mining asteroids, some humans will make money and help to generate industry, potentially manufacturing medicines to save lives on Earth. Concepts of risk are deeply endowed with cultural beliefs [41], which will affect decision-making in space because they define what is “risky” and how much risk is worth taking for a reward—money, fame, a successful mission, a sense of accomplishment, or an appreciation of new forms of beauty.

“Risk” will be examined here in two different conceptual frameworks that both have connections to the high level of human neuroplasticity. That trait allows variation in phenotypic expression, and it allows humans to make decisions about what to do and how to do it according to their environment, genetics, behavioral history, and social group. Humans can change decisions quickly, or slowly, even contravening their own good sense. In space, neuroplasticity may allow a crew to overcome the adverse effects of an environmental factor such as CO_2_ and thereby continue, for example, strict adherence to a protocol. Upsides and downsides to human neuroplasticity will need to be exploited for any specific successful mission.

The first framework we present is recent psychological research on graviception and decision-making. Perception of gravity mainly occurs in the vestibular system of the inner ear. However, perception of gravity is not confined to this finetuned apparatus as there are somatic graviceptors in the human torso [42].

The second context involves “risk neurogenes” (a term used in opposition to “spaceflight-resistant neurogenes”) and the human choice-reward apparatus seated primarily in the dopamine and serotonin systems of the brain.

### 4.1. Risk Perception and Decision-Making in a Weightless Analog

Gallagher Arshad, and Ferrè conducted research to determine how humans make choices in an experimental weightless analog environment [4,5]. They proposed that changing the orientation of the human body to a supine position, and thus changing the direction of gravitational pull, might change the choices that their experimental subjects made. Their thesis rested on the assumption that behavior is a “trade-off” between “exploiting old solutions and exploring new ones” [4] (p. 992). The design used a random number-generation task to illustrate whether the subject would make a decision to exploit familiarity (repeating digits) or explore new solutions (naming new digits). Results showed that subjects were less likely to generate random behaviors in a supine position than an upright position. They concluded, accordingly, that decisions in a weightless analog were less likely to be novel solutions. In summary, Gallagher and colleagues asked if a change in the human body’s orientation to gravity might affect routine vs. novel choice. Their conclusion was that “gravitational signals may shape the balance between exploitation and exploration, in favor of more stereotypes and routine responses” [4] (p. 989). If true, this has planning implications for humans in space.

This was indeed an innovative research design that should be followed up with other body postures. If one examines the postures of weightless astronauts on the ISS, most of them are positioned in the usual primate “relaxed fetal” position, with all four limbs crouching inward. The supine position is an unusual position for humans (unless they are well supported, as in bed), and if unsupported, it is often associated with falling and, therefore, alarm. This natural fear of falling might affect test results, producing more choices that are routine and safe.

Their research emphasized the importance of understanding decisions that involve novelty vs. familiarity. This is without question an important focus of future research since space crews will routinely encounter novel situations after (or punctuating) long periods of routine on lengthy space voyages. The heightened consequences of decision-making in space are apparent in Ferrè’s quotation of a Canadian astronaut, Chris Hadfield, which bears repeating here: “Most of the time, you only really get one try to do most of the critical stuff and the consequences are life or death” [5]. That summarizes well one aspect of the very different decision environment in space.

Managing the heightened anxiety felt when facing decisions with life and death consequences can become routine, however, as evidenced by soldiers in war, police on the beat, or the test pilots of multimillion-dollar jets. We know that anxiety can be managed in high-risk occupations. Anxiety, itself, is an important topic for future space research. It is an important aspect of these researchers’ principal question: does space inhibit or encourage risk-taking behavior? A broader research question would be: is space tolerable for some humans more than for others, and who are they?

Questions about risk-taking ability are important for space crews who find themselves in new places, with new challenges, and with a decision-making apparatus that we do not yet know well in microgravity. Investigators are asking whether human decision-making abilities remain the same, and if not, how they are changed, by how much, and with what implications for mission success.

### 4.2. Risk Neurogenes, Decision-Making, and Mission Planning

Popova et al.’s [3,43] work on “risk neurogenes” (in opposition to “spaceflight resistant neurogenes”) was mentioned when several good examples of research methodologies were discussed above. Here, their research results on rats and mice are summarized.

“Risk neurogenes” are some of the main regulators of brain neuroplasticity, including neurotransmitters and neurotrophic factors, which operate through the brain’s dopamine and serotonin systems, guiding reward and choice. Research results from mice in spaceflight identified which factors were affected by microgravity, and in which areas of the brain the factors operated. Some risk neurogenes were shown to have an altered function in mice after “long-term” spaceflight (a month, not years). Are they, therefore, logical targets for research on potential malfunctioning in humans? The authors wrote, “the investigations of the brain mechanisms underlying the development of behavioral disorders in spaceflight are at the very beginning, and the identification of risk genes for long-term spaceflight is one of the first steps towards understanding long-term spaceflight consequences for human behavior and brain functioning” [3] (p. 396). The study of risk neurogenes in humans could be an examination of some of the most fundamental aspects of what it means to be human, how the high human level of neuroplasticity might be regulated, and what happens to the regulation of brain functioning when humans venture off-world.

In summary, Popova et al. found that spaceflight affects both the main regulators of brain neuroplasticity—neurotransmitters (5-HT-serotonin and DA-dopamine) and neurotrophic factors (CDNF and GDNF, but not BDNF^1^). The brain’s response to spaceflight is different in different regions:

“Substantia nigra, striatum and hypothalamus are highly sensitive to the long-term spaceflight: in these brain areas spaceflight decreased the expression of both DA-related and neurotrophic factors genes. Since DA system is involved in the regulation of movement and cognition the data discussed in the review could explain dysfunction of locomotion and behavior of astronauts and direct further investigations to the DA system” [3] (p. 396).

These detailed research results will surely be augmented in the future by equivalent research on humans in space, to clarify human decision-making in space—its risks, rewards, and remedial steps, where needed and where possible.

However, let us re-state how critically important studies on the dopamine and serotonin reward systems are for understanding decision-making in space. More than a decade ago, there were models of the roles of dopamine and serotonin in decision-making, which explained the outcomes of value-based decision-making [44]. Indeed, Schultz speculated that the proper functioning of risk and reward systems originally guided how some thrived while others did not, and this formed the reason the brain evolved as it did [45]. The components of the dopamine reward system were listed in Popova et al.’s summary, and work has been ongoing to understand the roles of these specific components in overall decision-making. The basal ganglia, for example, is thought to be responsible for initiating behaviors, but not for determining the details of how they are carried out. Other components are involved, and they form a decision-making system [45].

The role of serotonin appears to be different, apparently guiding learning about bad decision outcomes, non-normative risk-seeking behavior, social choices involving affiliation and fairness, and cognitive appraisal of reinforcers when selecting between actions [44]. Homberg found serotonin to play an important role in decision-making, and she wrote, “based on 5-HT’s evolutionary role, I hypothesize that 5-HT integrates expected, or changes in, relevant sensory and emotional internal/external information, leading to vigilance behaviour affecting various decision making processes” [46] (p. 218).

If we examine Popova et al.’s diagrammed summary of influences from BDNF, GDNF, and CDNF^1^, we can see how “risk neurogenes” [3] (p. 401) could have very different effects on decision-making in space. Given this multiplicity of input avenues to decision-making, it becomes obvious why the careful parsing of factors that are susceptible to change in spaceflight, and those factors that are resistant to change, is so important. If Popova et al.’s results hold firm, then a careful delineation must be forthcoming that outlines how space can affect the decisions made by a crew.

## 5. Health Treatment and Remediation Programs

Harnessing neuroplasticity to manage performance degradations caused by the conditions of space (especially with regard to decision-making) may result in two main forms of action, with three periods for applying them: (1) using normal routines and medications, or (2) more novel genetic therapies and neuroprosthetics. The implementation periods are pre-flight preparation, in-flight application, and recovery procedures after missions. Routine and novel treatments will be treated separately in this article.

### 5.1. Routine Countermeasures

#### 5.1.1. Training and Exercise

Sgobba et al. [47] described the careful selection and comprehensive training of astronauts. Their rigor is essential for mission success and maintaining the performance and health of space crews. The regimes of various space agencies for preflight, in-flight, and postflight physical training (PT) were outlined by Loehr et al. [48]. The pre-flight physical training starts about 2 years before the mission, while the postflight reconditioning lasts a few weeks, depending on the individual crewmember’s needs. These include “neurovestibular, orthostatic, back/neck pain, coordination, balance/agility, aerobic, strength, endurance, power, and flexibility issues” [48] (p. A20). Postflight reconditioning is designed to return the astronaut to their pre-flight condition so they are ready for another mission. That pre-flight condition includes the ability to make swift and advantageous decisions.

Lessening the degradation experienced by the astronaut in-flight clearly puts a lower demand on their plasticity, and so experiments continue on how to select the best physical training programs. The aim is to maximize the effectiveness and minimize the duration of the PT so that crew members can spend more time on mission-oriented tasks. Accordingly, in the NASA SPRINT [Scheduling Planning Routing Intersatellite Network Tool] Project, high-intensity/low-volume training was favored over the previous default program of low-intensity/high-volume exercise since decrements in bone mineral density, muscle strength and endurance, and cardiorespiratory performance remained similar [49].

A recent modification applied to low-intensity exercise is the strategy of restricting blood flow to the exercised limb(s) [50]. The blood flow restriction (BFR) is achieved by a cuff applied to the limb, then inflated to compress the underlying vasculature. “The goal is to partially restrict arterial inflow to tissues distal to the tourniquet cuff while completely restricting venous out flow” [50] (p. 34). The local hypoxia produced by the blood flow restriction is returned to normal, pre-exercise levels within a few minutes. The whole process is efficient in time, has only minor on-board equipment needs, and produces effective results such as the downregulation of myostatin and several other genes involved in proteolysis. The gravity-like stress on the cardiovascular system could be particularly helpful for preparing an astronaut for the landing part of a mission. However, while promising, the BFR technique has only been applied to simulations of microgravity, particularly head-down tilt.

Regimes ranging from pharmaceutical supplements to advanced resistive exercise programs were reviewed by Tanaka et al. [51]. These included bisphosphonates, vitamin D_3_, and calcium. On the Mir space station, a combination of supplements, along with appropriate nutrition (see the next subsection), proved effective for avoiding bone mineral loss in the lower extremities.

Much more can be said about the myriad health-related benefits stemming from physical exercise, both on Earth and for space-related applications. These benefits are clearly related to human neuroplasticity since, among others, Sasmita et al. [1] found, for normal gravity conditions, that physical exercise “is a key treatment option to induce neurogenesis, correlating strongly with improved memory and attention as can be seen in rodents” [1] (p. 6). While some such neuroplasticity research is only concerned with curing symptoms, the enhancements needed for decision-making are implied.

#### 5.1.2. Diet and Other Mitigants

As with appropriate exercise, whether on Earth or in space, “adequate food consumption is central to maintaining health and performance” [52] (p. 1). Sirmons et al. then added, “this is especially true in demanding operational environments such as spaceflight, where body mass loss is common, and has physiological repercussions that include impaired cardiovascular performance, musculoskeletal losses, and oxidative stress.” They were investigating whether standard menus for breakfasts in space could be replaced with energy-dense, meal replacement bars (MRBs). The bars’ advantage is in reducing storage requirements in volume and weight, an important concern for space engineers. Not surprisingly, they found that even well-motivated and trained astronaut-quality individuals preferred their regular fare, though MRBs could be substituted in for short periods.

Neuroprotective foods and nutrients were considered in detail by Zwart et al. [53]. More broadly, both dietary and non-dietary countermeasures were comprehensively reviewed by Mhatre et al. [27] in relation to degradations in physical and cognitive performance during space missions. They also considered “crosscutting variables (e.g., biological sex) and [defined] critical features of spaceflight factors (e.g., gravity levels, radiation characteristics, flight durations)” (p. 909). Probiotics and prebiotics, along with multiple antioxidants, must feature in the diet. While adequate diet and exercise are important elements, it is the confluence of countermeasures that produced the desired results. That confluence calls for further studies, for they concluded: ” there remains a paucity of human studies using these alternative countermeasures and further investigation is needed” [27] (p. 927).

Non-consumable countermeasures include: improving sleep by taking an approach as simple as lowering the illumination to help the astronauts’ circadian rhythms; reducing the monotony in space by improving waketime schedules; shielding astronauts to mitigate their radiation exposure, including secondary exposure; applying some kind of artificial gravity, with gravity-loading skinsuits to reduce fluid redistribution in microgravity; even using virtual reality during exercise and to lower psychological stress; improvements to the all-important “support from home” communications. While these are all desirable on the ISS, they will be essential for deep-space missions, away from the radiation shield of the Earth’s magnetic field.

### 5.2. Novel Countermeasures

#### 5.2.1. Genetic Therapies

Popova et al.’s research [3] has been cited a number of times in relation to neurogenetics of brain plasticity. They carefully described dopamine and serotonin functions and depletions in space mice leading to behavioral disturbances, including to decision-making. They conclude that “after further detailed investigations the information concerning risk neurogenes will undoubtedly one day lead to new methods and pharmaceuticals that help to prevent the damaging effect of space travel on health of astronauts” [3] (p. 403).

A genetic therapy may be as simple as a zeitgeber, i.e., using some regular activity or phenomenon to keep the sleep-wake cycle or appetite on track [54]. Hitti et al. [55] outlined more direct therapeutic interventions:

“Gene therapy entails the delivery of genetic material to alter the expression of endogenous genes or to introduce exogenous genes [56]. To augment cellular function, gene therapy approaches may be used to provide a transgene. Commonly, a sequence encoding a wildtype human isoform of an enzyme is delivered. This therapeutic strategy may be used to provide a functional gene in patients with a mutated non-functional gene or an under-expressed gene. This technique can also be used to express proteins such as growth factors to enhance cell survival” (p. 16).

A number of gene therapy treatments are already approved by the U.S. Food and Drug Administration (FDA). Treatments include ones for leukemia, spinal muscular atrophy, and Parkinson’s disease (PD), which as a neurological disorder is particularly relevant to the treatment of dopamine deficiency in spaceflight. Hitti et al. [55] outlined over a dozen treatments, both with complete and ongoing trials, for augmenting dopamine levels in Parkinson’s patients. Currently, the putamen is the preferred injection site, but how to inject astronauts safely at that site is an important question. That is one reason why gene therapy is classified under “novel countermeasures”. Nonetheless, the growth factor GDNF has shown promise for providing a persistent trophic signal after a single infusion, which could be given during pre-flight preparations. Furthermore, Hitti et al. [55] indicated that neuromodulation via a gene therapy using AAV-GAD (adeno-associated virus (AAV)-glutamic acid decarboxylase (GAD)) treatment to the subthalamic nucleus may also be long-lasting. Therefore, dopamine enhancement via gene therapy is promising, but its application for astronauts is not ready yet.

Gene therapy for space applications goes a good deal further than Parkinson’s patients. Synthetic biology has been in development since the 1980s, and this is helping us to understand emergent properties that could enhance human adaptations to space conditions [57]. Precision gene-editing techniques such as CRISPR-Cas9 (clustered regularly interspaced short palindromic repeats-CRISPR-associated protein 9) [58] can be coupled with the identification of genes with valuable features such as radiation resistance, extra-strong bones, toleration of low oxygen levels, and enhanced memory. Additionally, studies in tissue engineering, stem cell applications, and 3D bioprinting are also being pursued with space missions in mind [59].

#### 5.2.2. Neuroprosthetics

Humans have used prosthetics for a long time. After canes, humans made eyeglasses and fashioned limbs for amputees. Neuroprosthetics go a step or two further, and substitute impaired motor, sensory, or cognitive functions. The cochlear implant is a familiar and successful example [60]. It invokes the brain computer interface (BCI), “a system that measures central nervous system activity and converts it into artificial output that replaces, restores, enhances, supplements or improves natural CNS output and thereby changes the ongoing interactions between the CNS and its external or internal environment” [61] (p. 3).

The potential for applying neuroprosthetics to decision-making, particularly in space, is apparent. If deep brain stimulation (DBS), a neuromodulatory device, can help to mitigate the effects of Parkinson’s disease with its dopamine deficiency [55], then it is worth considering the use of it as a countermeasure for the same deficiency experienced in space. Nevertheless, the potential is highly speculative, and related research raises substantial ethical issues [62], so it does not yet seem to have been considered in space-related medical journals. However, abundant new technologies for neuroprosthetics are being explored. In one example, Chitrakar et al. [63] focused on flexible and stretchable bioelectronics, and many clinical studies have been presented on potential neuroprosthetic technologies, e.g., Guidetti et al. [64], who wrote of adaptive deep brain stimulation (aDBS). The mental health of a space crew, an essential requisite for decision-making, could be stabilized by vagus nerve stimulation (VNS). Auditory tones [65] or more invasive VNS options [66] can produce more temporally targeted therapeutic effects than pharmacological treatments. Perhaps applications related to national security might lead the way into space [67]. It is not a stretch to imagine that they will become integrated.

There are less invasive neuroprosthetic techniques. A painless example is transcranial magnetic stimulation (TMS), which, with transcranial electrical stimulation (tES), has been used for a while to treat depression, schizophrenia, or as an analgesic, and for recovery from chronic motor dysfunction after stroke. Romanella et al. [68] reviewed the opportunities and challenges of noninvasive brain stimulation (NiBS) methods to support space exploration. Opportunities include: accelerated skill-training pre-flight; reduction of in-flight impairment in cognitive processes; support to cope with heavy physical demands such as EVAs; regulation of circadian rhythm disturbances. Finally, NiBS could be used to improve the plasticity on post-flight return to normal. Yet, the main challenges with implementing NiBS during space missions are the technological requirements, particularly those for transcranial magnetic stimulation because they currently involve large and heavy equipment. Helmets are in development that could be just as effective [68]. Transcranial electrical stimulation is more practical, if less comfortable since it causes some pain to the crew. The overall advantage of these less-invasive techniques is that they may be implemented faster than brain computer interface (BCI)- and deep brain stimulation (DBS)-based applications.

Overall, these novel countermeasures are speculative, but they promise a great deal in harnessing neuroplasticity to improve decision-making in space environments.

## 6. Discussion

### 6.1. Dual Role of Neuroplasticity as a Foundation for Decision-Making

Early research results suggest that neuroplasticity provides a necessary foundation for *both* neurological changes *and* neurological recovery, i.e., for phenotypic “adaptation” in both negative and positive senses. Hupfeld and colleagues [39] wrote of “dysfunction” and “adaptive plasticity”. Importantly, they summarized a growing impression from space research as follows:

“Research over the past decade has demonstrated two co-occurring patterns of spaceflight effects on the brain and behavior: dysfunction and adaptive plasticity. Evidence indicates the spaceflight environment induces adverse effects on the brain, including intracranial fluid shifts, gray matter changes, and white matter declines. Past work also suggests that the spaceflight environment induces adaptive neural effects such as sensory reweighting and neural compensation” [39] (p. 176).

Our growing suspicion is that neuroplasticity forms a foundation that, unsurprisingly, provides a means for understanding and managing neurological changes through pharmaceutical or environmental means, by applying neuromodulation or neuroprostheses, or using much simpler dietary or exercise protocols. Popova et al. framed the investigative situation with the following, challenging words:

“The effects of long-term microgravity exposure on the brain plasticity are the milestone problem of space neuroscience. The investigations of the brain mechanisms underlying the development of behavioral disorders in spaceflight are at the very beginning, and the identification of risk genes for long-term spaceflight is one of the first steps towards understanding long-term spaceflight consequences for human behavior and brain functioning” [3] (p. 396).

### 6.2. Very Long Space Voyages May Depend on the Human Evolutionary Past

Many types of metabolic and neurological changes could conceivably impact human cognition and decision-making in space. While this appears alarming at first, the high level of human neuroplasticity may allow controlled recovery in weightless and partial-g environments. Humans appear to be ideally suited among mammals for spaceflight and recovery from the changes wrought by weightlessness, radiation, and heightened carbon dioxide levels on their neurological systems. The evolutionary origins of neuroplasticity lie in the extension of anthropoid juvenile features into adulthood, leaving adult humans with extraordinary flexibility of the mind and body. Bruner and Gleeson wrote:

“Human prosthetic capacity is largely enhanced by the remarkable plasticity of our cortical system, and by the high level of creativity and explorative innovation of our species. Both features (neural plasticity and explorative behavior) are primarily associated with juvenile life stages and have been enhanced by extension of the juvenile period in humans” [69] (p. 2).

The authors hypothesized that the origin of human neuroplasticity lay in the interaction and amalgamation of body cognition, visuospatial integration, technological extension, and changes in the parietal cortex that occurred during evolution of the human line [69]. Thanks to the flexibility inherent to that combination of features, humans arrived in the modern era prepared for the physical and mental vicissitudes of spaceflight, and for both life and work at gravities lower (and perhaps higher) than Earth’s gravity, where we have evolved. The research questions now become: how much neurological change occurs with different combinations of environmental factors (radiation, weightlessness, increased CO_2_, others), and how much recovery is naturally available to the human species, or available through medical intervention, or a combination of both?

Human neuroplasticity likely emerged at a high level to assist with the mastery of higher neurocognitive skills, such as the skills maximized in spaceflight—dual- and triple-tasking, decision-making in rapidly changing gravities, use of concurrent and changing symbolic systems, unexpected and sequential maneuvers in three dimensions, and evaluation of data from multiple complex sources—both by the crew in space and also the support staff on Earth, who may be removed from them by varying time delays.

Paleoneurologists have discussed human neuroplasticity, not as an unfortunate negative byproduct of evolution, but as an advantage that helps humans learn different cultures and skills [69,70]. The resulting language and social skills are not to be minimized for crews who must live in confined quarters for lengthy periods and get along with their crewmates. Eventually, these skills will support First Contact, perhaps soon or perhaps hundreds of years from now. Those skills will still be important then.

The human species may be sufficiently flexible to absorb neurological changes and work around them with medical support. It has done so in so many other challenging situations on Earth, such as at high altitudes, at the depths of oceans, on the battlefield, and in sickness and injury. It may be unwise to assume that human decision-making is dangerously impaired without firm, direct proof over substantial periods. These research settings may not be available until a space mission is underway. So, how can potential dangers be tested? That question requires creative research designs and testing situations not yet available.

Humans off-world will be aided by artificial intelligence and robotic devices for construction, exploration, mining, and mapping. Many low-level decisions will be made by AI, but humans should monitor all final decisions. An important medical question is: which aspects of decision-making are most in danger? Research results point in a variety of directions, for example, Clément et al. [13] reported that rodents exposed to HZE (high-energy charged particles, a component of galactic cosmic radiation) at doses expected on a Mars mission had deficits in learning and memory, including novel object recognition and spatial memory, which depend on the hippocampus. That surely warrants follow-up with research on humans. There continues to be alarm in reaction to studies showing that the “brain changes” after “long spaceflights” of five and a half months, which affect cognition [11]. In the future, when flights extend to years—over three years for a voyage to Mars, and five to seven years to reach the moons of Jupiter and Saturn—the effects may be substantial without, or even with, medical intervention, if indeed treatment is needed (in all instances, or needed for all crew, or continuously for everyone). Will this medical support make the difference for human decision-making? Can neuroplasticity assist human tolerance of periods of higher-than-normal CO_2_ on spaceflights? Can decision-making be assisted by forms of neuromodulation or neuroprosthetics [71], or by training, or genetic surgeries, to ensure that human decision-making remains sound? Test pilots have long been trained to manage short periods of black-out when under the stress of several gravities. Will space crew need comparable training? The understanding of neuroplasticity’s dangers and advantages should point the way toward identifying the greatest causes of concern and the greatest advantages neuroplasticity has to offer for the space traveler.

## 7. Conclusions

This article has provided an exploratory review of research on neuroplasticity relating to humans in space and their capacity to maintain advanced decision-making skills, which will be required for mission success on long space journeys. An array of changes to the human brain occurs during spaceflight, and understanding their nature will be essential for long space journeys and settlement of the solar system. Looking ahead, we need to develop a system of “comprehensive monitoring and countermeasure strategies for future long-duration space exploration” [72] (p. 1).

We are developing a broad understanding of neuroplasticity as a “dual-valence” capacity that occurs at a very high level in modern humans. Details presented in this analysis set the foundation for future results that may enable the true “harnessing” of neuroplasticity, both to maximize its benefits and control its deficits for human decision-making in space.

Decision-making by humans remains a complex process, the details of which vary from the genetic to the group level. Neuroplasticity is emerging as a basis for assessing the adequacy of decision-making processes in space. Much is unknown about humans in space, and yet there is the possibility that human neuroplasticity may enable the return of humans to a functional state after deconditioning in space. Beyond this, there is hope that humans will be able to maintain their extraordinary decision-making capabilities—both singly and together in groups—on the long space journeys that lie ahead of us.

Through future research that builds on the knowledge generated by the researchers cited in this paper, and related research from their colleagues, neuroplasticity’s effects on human decision-making—a fascinating aspect of space science—will soon come to be better understood.
